# Evidence-Based Physical Therapy Practice in the State of Kuwait: A Survey of Attitudes, Beliefs, Knowledge, Skills, and Barriers

**DOI:** 10.2196/12795

**Published:** 2019-06-07

**Authors:** Hesham N Alrowayeh, Ali J Buabbas, Talal A Alshatti, Fatemah M AlSaleh, Jawad F Abulhasan

**Affiliations:** 1 Physical Therapy Department Faculty of Allied Health Sciences Kuwait University Jabriya Kuwait; 2 Department of Community Medicine and Behavioral Sciences Faculty of Medicine Kuwait University Jabriya Kuwait; 3 Department of Pharmacy Practice Faculty of Pharmacy Kuwait University Jabriya Kuwait; 4 Shikhan Alfaresi Rehabilitation Center Physical Therapy Department Ministry of Health Sulaibikhat Kuwait

**Keywords:** physical therapy practice, evidence-based practice, attitudes, knowledge

## Abstract

**Background:**

Evidence-based practice (EBP) is necessary to improve the practice of physical therapy. However, a lack of knowledge and skills among physical therapists and the presence of barriers may hinder the implementation of EBP in the State of Kuwait.

**Objective:**

The objectives of this study were to extensively (1) investigate attitudes toward EBP, (2) assess the current level of knowledge and skills necessary for EBP, and (3) identify the barriers to EBP among physical therapists in the State of Kuwait.

**Methods:**

The following methods were used: (1) a previously validated self-reported questionnaire and (2) a face-to-face semistructured interview. The questionnaire, which was distributed to 200 physical therapists, examined the attitudes and beliefs of physical therapists about EBP; the interest in and motivation to engage in EBP; educational background, knowledge, and skills related to accessing and interpreting information; the level of attention to and use of the literature; access to and availability of information to promote EBP; and the perceived barriers to using EBP. The interview explored the factors that promote or discourage EBP. Descriptive statistics and logistic regression analyses were used.

**Results:**

Of the 200 nonrandomly distributed questionnaires, 92% (184/200) were completed and returned. In general, the physical therapists had positive attitudes, beliefs, and interests in EBP. Their educational background, knowledge, and skills related to assessing and interpreting information were well-founded. The top 3 barriers included insufficient time (59.2%, 109/184), lack of information resources (49.4%, 91/184), and inapplicability of the research findings to the patient population (40.7%, 75/184).

**Conclusions:**

EBP lacks support from superiors at work. Thus, identifying methods and strategies to support physical therapists in adopting EBP in the State of Kuwait is necessary.

## Introduction

### Background

There is no doubt that evidence-based practice (EBP) is essential to improve physical therapy (PT) practice, as this strategy ties high-quality research evidence with the clinical expertise of physical therapists (PTs) and patient preferences to achieve the best clinical decision and PT care. EBP can also minimize the misuse, overuse, and underuse of health care services [1].

Globally, numerous studies have investigated the attitudes, skills, knowledge, and barriers to practice based on evidence among health care providers [2-5] including PTs [6-17]. Generally, PTs had positive attitudes toward EBP [6-13], with the majority of PTs believing that the use of research evidence is necessary to their practice [6,8-10,15]. The required skills and knowledge included the ability to phrase and ask a clinical question; search the literature using Boolean operators; and find, read, and critically evaluate the research findings using statistical knowledge to select the best and up-to-date evidence at any stage of PT care [6-8,10,13-16]. Lack of time was the most commonly reported barrier [6-8,10,11,15-17]. Other barriers included the lack of skills in identifying and critically evaluating research evidence, insufficient administrative support, insufficient access to evidence, inability to apply research findings to specific patient populations, and insufficient teaching in previous education.

In the State of Kuwait, PT care is provided and financed primarily by the government through the Ministry of Health (MoH). Approximately, 700 PTs are working in various specialties in various general and specialized hospitals distributed in 6 health regions: Alahmadi, Alfarwaniya, Aljahra, Alsabah, Hawally, and Alasema [18]. The majority of PTs in the workforce are non-Kuwaiti and are foreign-trained [18]. Foreign-trained PTs sit for a licensure exam before registering with the Kuwait Medical Licensing Authority (KMLA), whereas Kuwait-trained PTs only register with KMLA. Access to PT care is not direct and patients are referred from primary or secondary care. Similar to the health services, PT education is offered mainly through the government university. This one and only university has been offering a bachelor’s degree since 1982.

Strong training in EBP is not included in the university curriculum nor is it practiced among PTs in the MoH. This was evident in the literature that examined the extent of using research evidence as the basis for PT practice in Kuwait [19]. The study included Kuwaiti musculoskeletal PTs only and found that the current PT practice was more reliant on the basic knowledge acquired during undergraduate education, with minimal use of research evidence to inform the choice of the therapy technique. Despite these findings, PTs in Kuwait are trying to shift toward EBP [20]. However, they apparently lack the knowledge and skills necessary to practice PT based on evidence.

On the basis of the literature, a comprehensive in-depth investigation of the attitudes, the current level of knowledge and skills, and the barriers related to EBP among PTs is lacking in the State of Kuwait. Previous studies [19,20] touched superficially and indirectly on this topic and did not include all practicing PTs, Kuwaiti and non-Kuwaiti in various PT specialties. Needless to say, the findings from this study might help to (1) encourage the use of EBP among PTs, (2) identify methods to support PTs in adopting EBP, and (3) develop guidelines and implementation strategies for EBP in the State of Kuwait.

### Objectives

The objectives of this study were to (1) investigate attitudes toward EBP, (2) assess the current level of knowledge and skills related to EBP, and (3) identify the barriers to practicing EBP among PTs in the State of Kuwait.

## Methods

Quantitative and qualitative methods were used to collect the data over a 3-month period (September to December 2016) to achieve the study objectives. The study was approved by the institutional review boards at both Kuwait University and the MoH in the State of Kuwait. The participants and instruments used for each method are described below.

### Participants

For the quantitative analysis, a convenient sample of 200 PTs received an invitation to participate in this study from approximately 660 eligible PTs currently working in the government hospital located in all governorates of the State of Kuwait. Participants were recruited if they had at least 1 year of clinical experience and were currently working in government hospitals and clinics. In addition, participants were recruited if they could read and understand English, which was the questionnaire language.

For the qualitative analysis, the directors of 8 PT departments were interviewed from the following health regions: Alfarwaneya, Hawally, Alasema, Aljahraa, Alahmadi, and Alsabah. A total of 6 directors worked in general care and 2 directors worked in specialized care. In addition, the head director of the PT departments at the MoH was interviewed. Participants had at least 15 years of clinical experience and at least 5 years of administrative experience. Directors were included in the study to explore the liaisons between the higher management and the working PTs. In particular, the interviews with PT directors would provide a good understanding about the current situation of PT and EBP, including the facing difficulties, together with the ways to overcome them practically and administratively.

### Instruments

#### Questionnaire

For the quantitative analysis, a self-reported questionnaire was used in this study. The questionnaire was previously developed, wherein the content validity and reliability (Intraclass Correlation Coefficient (1, K) ranged from .37 to .90) of the questionnaire were established [6]. The questionnaire comprised 32 items. These items examined the PTs’ (1) attitudes and beliefs about EBP (items 1, 2, 4, and 6 to 11); (2) interest in and motivation to engage in EBP (items 3 and 5); (3) educational background, knowledge, and skills related to accessing and interpreting information (items 25 to 31); (4) level of attention to and use of the literature (items 12 to 14); (5) access to and availability of information to promote EBP (items 18, 19, and 21 to 23); and (6) perceived barriers to using EBP (item 32). The participants rated their attitudes, beliefs, education, knowledge, and skills related to EBP using a 5-point Likert scale with responses varying from *strongly disagree* to *strongly agree*. Items related to access to information required *yes/no* responses. In addition, demographic and practice data were collected, including the age, gender, nationality, education degree, years of experience, area of specialty, work environment, and patient load of the participant, as well as their contribution to research.

#### Interview

For the qualitative analysis, face-to-face semistructured interviews were conducted with the PT directors. The interview used questions that were developed by the research team in a previous study [5]. The questions were reviewed for relevance to this study. No changes were made, and the questions focused on 3 main themes: (1) background on EBP; (2) opinions on implementing EBP; and (3) current barriers and facilitators to adopting EBP in the PT field. See Multimedia Appendix 1 for interview questions.

#### Procedures

The cross-sectional survey was conducted by distributing the questionnaires to PTs at their workplace using a convenience sampling technique. The questionnaire, as well as the invitation letter and consent form, was distributed and collected by the fifth author over the period of a week. The letter explained the purpose of the study and assured the participant confidentiality of their responses.

Separate interviews with the head director of the PT departments at the MoH and the director of each PT department were arranged in advance. The interviews were conducted by the second author. They were transcribed verbatim. File notes were taken during and after the interview. The notes were typed out directly after completion of the interviews to ensure accurate documentation of all information.

#### Data Analyses

Descriptive statistics, including number and percentage of the demographical data of all participants were calculated. Logistic regression tests were used to assess the association between the demographics and research-related items (attitudes and beliefs about EBP; interest in and motivation to engage in EBP; educational background, knowledge, and skills related to accessing and interpreting information; the level of attention to and use of the literature; access to and availability of information to promote EBP; and perceived barriers to using EBP). Odds ratios and 95% CIs were calculated if a significant association was found. Statistical significance was evaluated at alpha=.05; statistical analyses were conducted using SPSS (version 24, SPSS Inc).

A content analysis approach was followed to analyze the interview data. Data from the open- and close-ended questions were transcribed verbatim to enable qualitative data analysis. A list of codes was initially developed based on the content of the semistructured interviews. Additional codes and subcodes were then added as new issues were raised by the participants during the interviews. The coding process was done manually wherein the transcripts were read thoroughly and each text segment was assigned a code or subcode. All transcripts were systematically searched for similar codes or subcodes using an iterative approach, in which the constant comparison method was used throughout the data management [21]. By this method, the data were constantly revisited after initial coding until it was clear that no new codes or subcodes were emerging.

The coding was performed by 2 independent researchers who have expertise in qualitative data analysis. The codes and subcodes by both researchers were compared for similarities and differences for the first 2 transcripts, and no major differences were identified. Therefore, the interrater reliability was not calculated.

## Results

### Results From the Questionnaire

#### Participation Rate

A total of 184 PTs completed and returned the questionnaires. The return rate was 92% (184/200). No questionnaire was returned with no less than 95% of the total questions answered. The time taken to complete the questionnaire was 5 to 10 min.

#### Participant Description

The sociodemographic data of the PTs are shown in Table 1. The results show that the majority of the study population were non-Kuwaiti (58.8%, 106/180) and female (60%, 108/180). Almost 29% (51/180) of the PTs were ranked as PT specialists, with the majority (73.2%; 131/179) holding entry-level degrees. More than half of the participants (62.6%, 109/174) had more than 10 years of clinical experience (mean 14.0 years). More than one-third of the PTs worked in general hospitals (38.4%, 70/182) or in rehabilitation hospitals (37.9%, 69/182). Most of the respondents worked for a minimum of 30 hours per week.

**Table 1:** Study participant demographics.

Demographics		n^a^ (%)
**Gender**		
	Male	72 (40)
	Female	108 (60)
**Age (years)**		
	20-29	42 (23)
	30-39	79 (43.2)
	40-49	50 (27.3)
	50+	12 (6.5)
**Education**		
	Bachelor of Science degree	131 (73.2)
	Master of Science degree	48 (26.8)
**Professional rank**		
	Junior PTs^b^ practitioner	28 (15.6)
	PTs practitioner	19 (10.6)
	Senior PTs practitioner	42 (23.3)
	PTs specialist	51 (28.3)
	Senior PTs specialist	30 (16.7)
	Superintendent PTs	10 (5.6)
**Working hours per week**		
	<10	22 (12.8)
	10-19	5 (2.9)
	20-29	38 (22.1)
	30-39	62 (36)
	40+	45 (26.2)
**Work settings**		
	General hospital	70 (38.4)
	Rehabilitation hospital	69 (37.9)
	Specialized hospital	43 (23.6)
**Participation in continuous education**		
	Yes	138 (75)
	No	46 (25)
**Nationality**		
	Kuwaiti	75 (41.4)
	Unidentified	19 (10.5)
	Egyptian	17 (9.4)
	Filipino	3 (1.7)
	Indian	55 (30.4)
	Saudi	7 (3.9)
	Iraqi	3 (1.7)
	Lebanese	1 (0.6)
	Palestinian	1 (0.6)
**Nature of work**		
	Patient care	58 (32.2)
	Administration	177 (97.8)
	Supervising students	58 (32)
	Teaching	31 (17.1)
**Professional experience (years)**		
	0-5	33 (19)
	6-10	32 (18.4)
	11-15	38 (21.8)
	16-20	38 (21.8)
	21+	33 (19)
**Patient load per day**		
	1-7	92 (50.8)
	8-12	69 (38.1)
	12+	11 (11)
**Areas of specialty**		
	Neurology	34 (19.1)
	Cardiopulmonary	28 (15.7)
	Pediatrics	29 (16.3)
	Orthopedics	87 (48.9)

^a^Column values do not always add up to the total number of questionnaire respondents because of missing data.

^b^PTs: physical therapists.

#### Attitudes, Beliefs, and Interests

**Figure 1:**
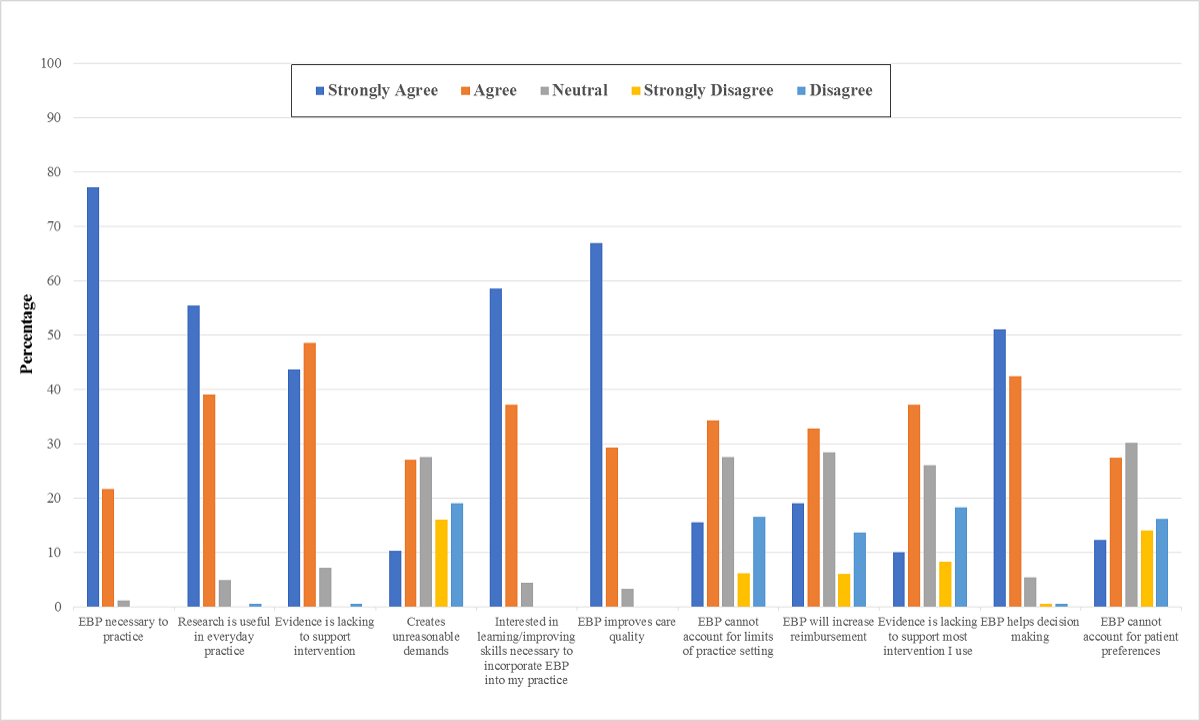
Self-reported attitudes and beliefs about evidence-based practice (EBP).

The distribution of the data on attitudes, beliefs, and interests of the PTs about EBP is shown in Figure 1. Most of the PTs strongly agreed that the application of EBP was necessary to their practice (77.2%); EBP can improve the quality of patient care (66.9%); the literature and research findings are useful in everyday practice (55.4%); EBP can help with decision making (51.1%); and more evidence should be used in daily practice (48.6%). The majority of PTs indicated that they were strongly interested in learning or improving the skills necessary to incorporate EBP into their practice (58.5%). Some believed that strong evidence is not available to support most of the interventions they used with their patients (37.2%); EBP does not take into account the limitations of their clinical setting (34.3%); the adaption of EBP may place unreasonable demands on them (27.0%); and it cannot account for patient preferences (27.4%).

Significant associations were identified between some demographic factors (age, working hours, and work settings) and attitude and beliefs of the PTs (Table 2). In particular, PTs working in general hospitals believed more than those working in rehabilitation or specialized hospitals that research evidence supporting most of the interventions they used was lacking. In addition, the PTs working for longer hours believed more strongly than the PTs working shorter hours that EBP places unreasonable demands on them. Furthermore, older respondents and male respondents were more likely to hold this belief.

#### Education, Knowledge, and Skills

The majority of PTs either agreed or strongly agreed that they learned the foundations of EBP as a part of their academic program (79%); they received formal training in search strategies (62.7%); they were familiar with Web-based search engines (73.8%); they received formal training in critical appraisal (56.8%); they were confident in their ability to critically review the professional literature (70.5%); and they were confident in their ability to find research relevant to their clinical questions (81.5%).

**Table 2:** Factors (demographics) associated with beliefs about evidence-based practice. The number of respondents varies because of missing data.

Attitude or belief and factor			Odds ratio (95% CI)	Model *P*^a^ value	Model *R*^2b^
**Item #4: Creates unreasonable demands**					
	**Age (years; n=173)**			.004	.100
		20-30	0.091 (0.02-0.422)		
		31-40	0.194 (0.045-0.831)		
		41-50	0.286 (0.07-1.164)		
		51+	Reference		
	**Gender (n=170)**			.004	.064
		Male	2.517 (1.326-4.779)		
		Female	Reference		
	**Working hours/week (n=162)**			.045	.080
		<10	0.596 (0.201-1.771)		
		10-19	0.256 (0.088-0.746)		
		20-29	1.917 (0.289-12.719)		
		30-39	0.902 (0.402-2.024)		
		40+	Reference		
**Item #9: Evidence is lacking to support most intervention I use**					
	**Work settings (n=178)**			.003	.086
		General hospital	1.034 (0.476-2.247)		
		Rehabilitation hospital	0.342 (0.154-0.762)		
		Specialized hospital	Reference		

^a^In logistic regression, one level of the independent variable serves as a reference against which the odds of the other levels occurring are determined.

^b^Nagelkerke *R*^2^.

**Figure 2:**
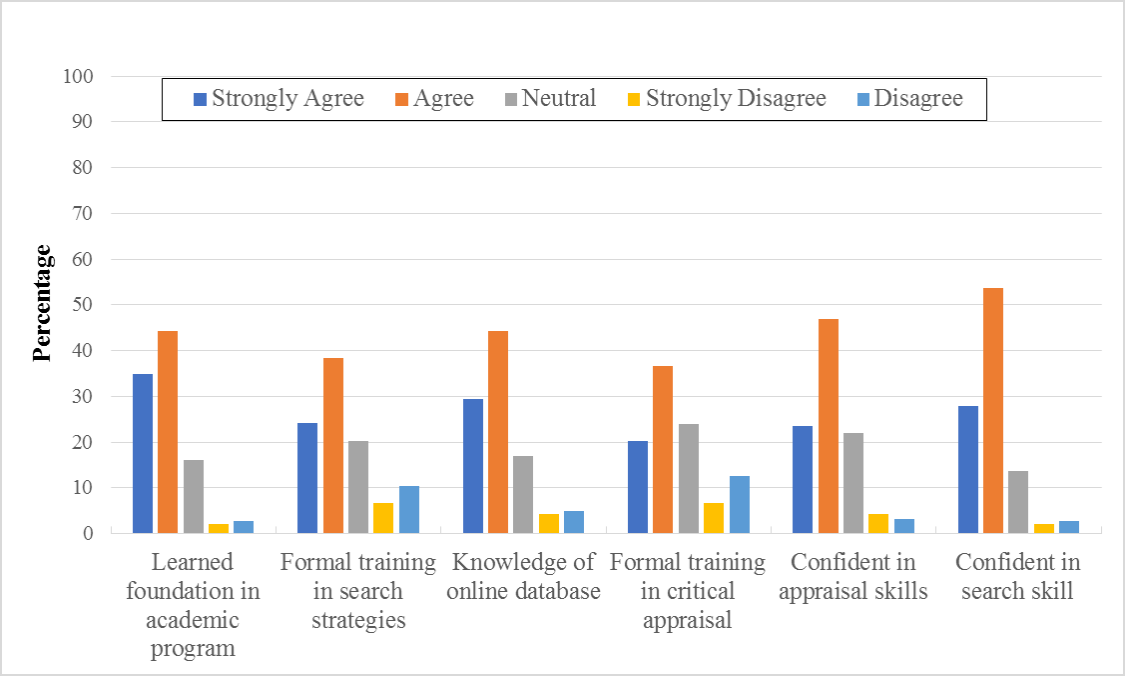
Self-reported education, knowledge, and skills.

Figure 2 shows the distribution of the education, knowledge, and skills of the PTs about EBP. The results showed that the education, work settings, and area of specialty were significantly associated with the educational background of the PTs and their knowledge and skills related to accessing and interpreting information (Table 3). For example, PTs with Bachelor of Science (BSc) degrees had received more training in search strategies and critical appraisal and were more confident in search skills than PTs with Master of Science (MSc) degrees. In addition, participants with BSc degrees were more familiar with the medical search engines and had learned more about the foundations of EBP compared with those with MSc degrees. PTs working in general hospitals had received more training in search strategies and the critical appraisal of research and were more confident in their search skills than PTs working in rehabilitation or generalized hospitals. PTs working in general hospitals were also more familiar with the medical search engines. Finally, PTs practicing neuro-rehabilitation were more confident in their appraisal skills than PTs practicing other specialties.

**Figure 3:**
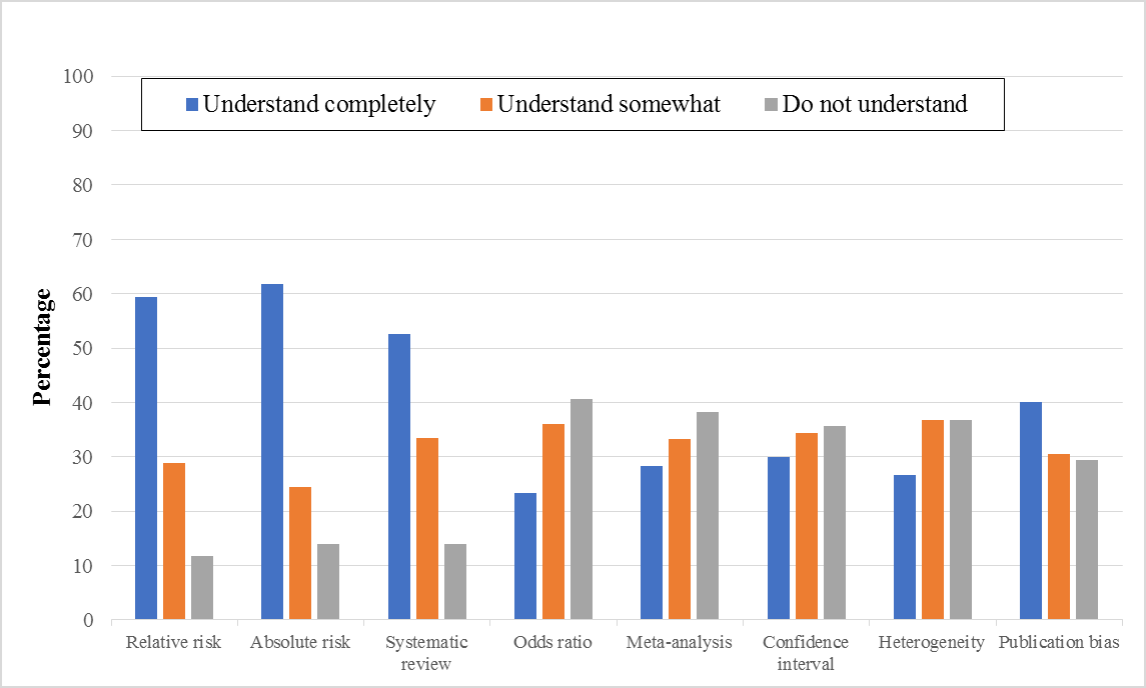
Self-reported knowledge of specific terms.

The results reveal that PTs had diverse knowledge with regard to the terms related to EBP. The PTs largely understood the following terms: *relative risk* (59.4%), *absolute risk* (61.7%), and *systematic review* (52.5%). However, many PTs did not understand the following terms: *publication bias* (40%), *odds ratio* (40.6%), *meta-analysis* (38.3%), *confidence interval* (35.6%), and *heterogeneity* (36.7%; Figure 3). The PTs with BSc degrees were more likely to understand the terms *odds ratio*, *meta-analysis*, *confidence interval*, *heterogeneity*, and *publication bias* than the PTs with MSc degrees (Table 4).

**Table 3:** Factors (demographics) associated with education, skills, and knowledge necessary for evidence-based practice. The number of respondents varies because of missing data.

Attitude or belief and factor			Odds ratio (95% CI)	Model *P*^a^ value	Model *R*^2^^b^
**Item #25: Learned foundation in academic program**					
	**Education (n=176)**			.02	.047
		BSc^c^	0.335 (0.122-0.917)		
		MSc^d^	Reference		
	**Gender (n=177)**			.046	.035
		Male	2.19 (0.988-4.854)		
		Female	Reference		
**Item #26: Formal training in search strategies**					
	**Education (n=177)**			.004	.061
		BSc	0.343 (0.157-0.747)		
		MSc	Reference		
	**Work settings (n=180)**			<.001	.137
		General hospital	0.859 (0.353-2.087)		
		Rehabilitation hospital	0.225 (0.096-0.530)		
		Specialized hospital	Reference		
**Item #27: Knowledge of Web-based database**					
	**Education (n=178)**			<.001	.098
		BSc	0.190 (0.064-0.565)		
		MSc	Reference		
	**Gender (n=179)**			.01	.053
		Male	2.535 (1.212-5.304)		
		Female	Reference		
	**Work settings (n=181)**			.03	.055
		General hospital	0.713 (0.265-1.920)		
		Rehabilitation hospital	0.334 (0.130-0863)		
		Specialized hospital	Reference		
**Item #28: Formal training in critical appraisal**					
	**Education (n=178)**			.02	.041
		BSc	0.438 (0.215-0892)		
		MSc	Reference		
	**Work settings (n=181)**			<.001	.113
		General hospital	1.250 (0.557-2.807)		
		Rehabilitation hospital	0.007 (0.150-0.735)		
		Specialized hospital	Reference		
**Item #29: Confident in appraisal skills**					
	**Gender (n=179)**			.003	.068
		Male	2.819 (1.378-5.768)		
		Female	Reference		
	**Work settings (n=181)**			.002	.092
		General hospital	0.401 (0.246-1.754)		
		Rehabilitation hospital	0.004 (0.096-0.631)		
		Specialized hospital	Reference		
	**Area of specialty (n=177)**			.008	.092
		Neurology	5.143 (1.680-15.740)		
		Cardiopulmonary	2.815 (0.946-8.376)		
		Orthopedics	0.002 (1.712-10.257)		
		Pediatrics	Reference		
**Item #30: Confident in search skills**					
	**Education (n=178)**			.02	.046
		BSc	0.317 (0.105-0.955)		
		MSc	Reference		
	**Gender (n=179)**			.002	.085
		Male	3.899 (1.522-9.984)		
		Female	Reference		

^a^In logistic regression, one level of the independent variable serves as a reference against which the odds of the other levels occurring are determined.

^b^Nagelkerke *R*^2^.

^c^BSc: Bachelor of Science degree.

^d^MSc: Master of Science degree.

**Table 4:** Factors (demographics) associated with understanding of specific terms. The number of respondents varies because of missing data.

Terms and factor			Odds ratio (95% CI)	Model *P*^a^ value	Model *R*^2^^b^
**Odds ratio**					
	**Gender** **(n=176)**			.04	.033
		Male	1.948 (1.035-3.667)		
		Female	Reference		
**Meta-analysis**					
	**Education (n=175)**			<.001	.095
		BSc^c^	0.248 (0.107-0571)		
		MSc^d^	Reference		
	**Gender** **(n=176)**			.014	.046
		Male	2.221 (1.159-4.259)		
		Female	Reference		
**Confidence interval**					
	**Education (n=175)**			<.001	.099
		BSc	0.232 (0.097-0.558)		
		MSc	Reference		
	**Gender (n=175)**			.05	.030
		Male	1.905 (0.991-3.661)		
		Female	Reference		
	**Work settings (n=180)**			.001	.109
		General hospital	0.697 (0.282-1.722)		
		Rehabilitation hospital	0.235 (0.098-0.565)		
		Specialized hospital	Reference		
**Heterogeneity**					
	**Education (n=175)**			.003	.069
		BSc	0.314 (0.140-0.704)		
		MSc	Reference		
**Publication bias (n=175)**					
	**Education**			<.001	.121
		BSc	0.160 (0.054-0.475)		
		MSc	Reference		
	**Gender (n=176)**			.003	.071
		Male	2.929 (1.404-6.111)		
		Female	Reference		

^a^In logistic regression, one level of the independent variable serves as a reference against which the odds of the other levels occurring are determined.

^b^Nagelkerke *R*^2^.

^c^BSc: Bachelor of Science degree.

^d^MSc: Master of Science degree.

**Figure 4:**
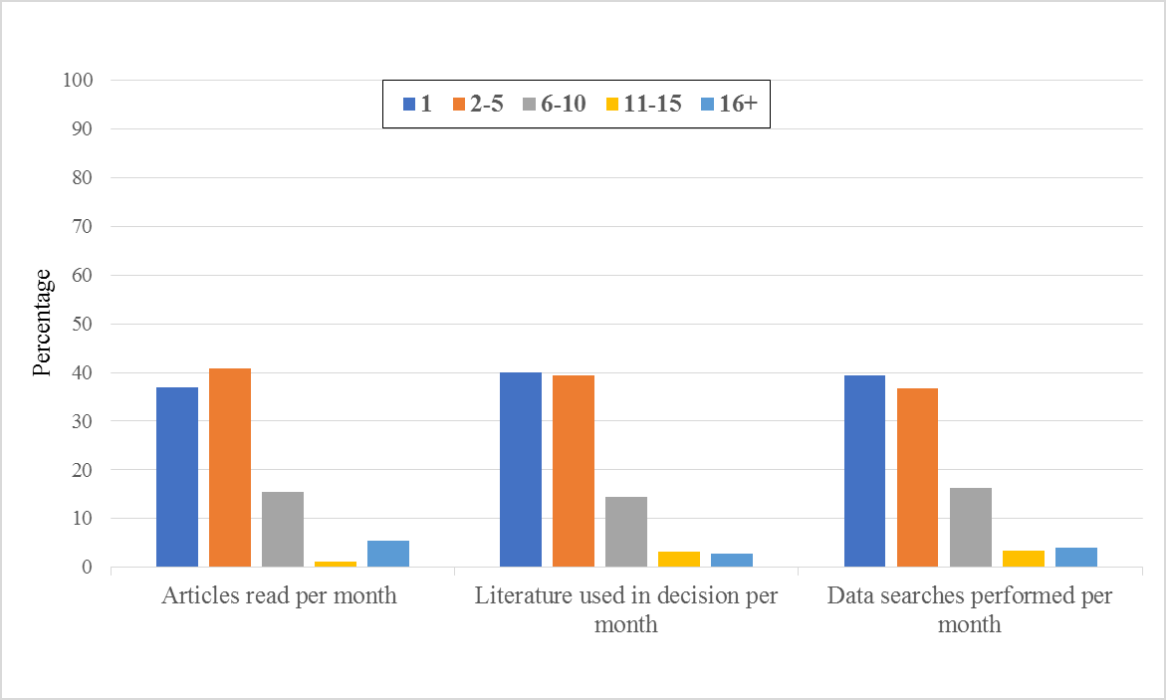
Self-reported attention to the literature.

#### Attention to the Literature

On average, per month, the majority of the PTs read 1 to 5 articles related to their clinical practice (77.9%); used 1 to 5 articles in the process of clinical decision making (79.4%); and performed 1 to 5 data searches for practice-relevant literature (76.2%; Figure 4).

#### Access to and Availability of Practice Guidelines and the Literature

**Figure 5:**
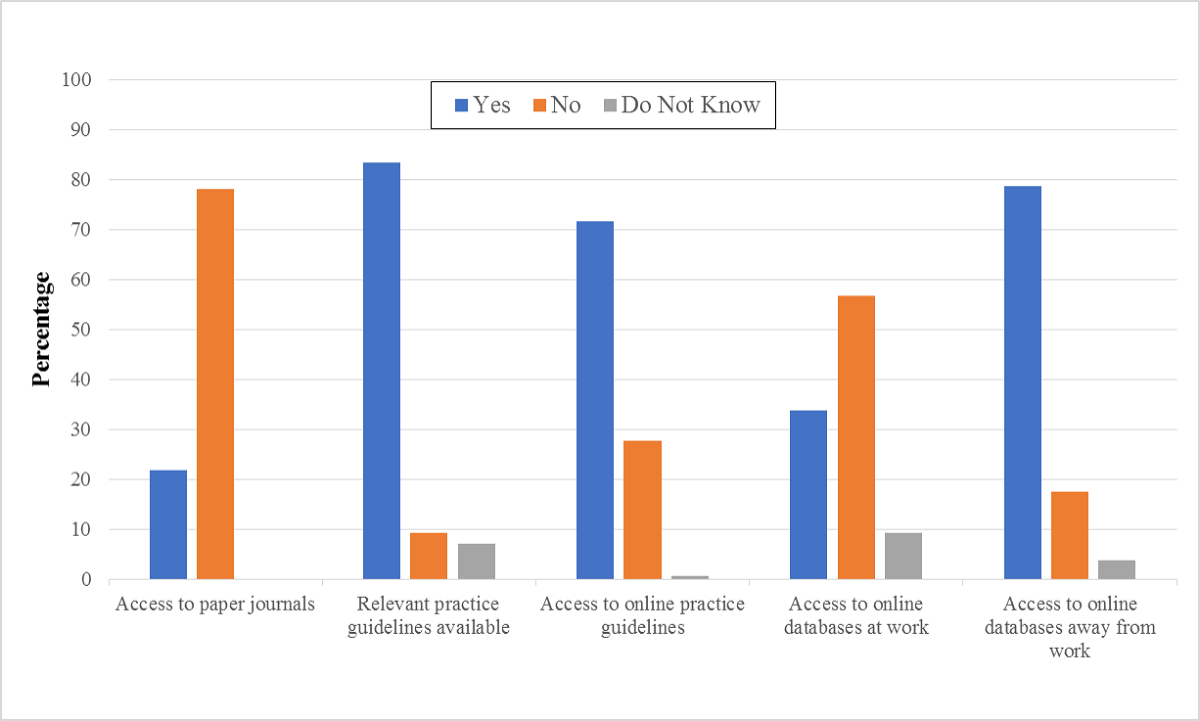
Self-reported access to and availability of literature.

At work, practice guidelines were available to the majority of PTs (83.5%) and were available on the Web (71.7%). However, access to current paper journals (78.1%) and/or relevant databases (56.8%) was not available to the PTs at their work. Therefore, these individuals mostly accessed the relevant databases away from their work (78.7%; Figure 5).

The work settings and hours, age, gender, and area of specialty were significantly associated with the access to and availability of practice guidelines and the literature (Table 5). For instance, access to paper journals and relevant Web-based databases at work was more possible among PTs working in general hospitals than access among those working in rehabilitation or specialized hospitals. Access to relevant databases outside of work was more likely among middle-aged PTs practicing orthopedic PT.

#### Barriers

**Figure 6:**
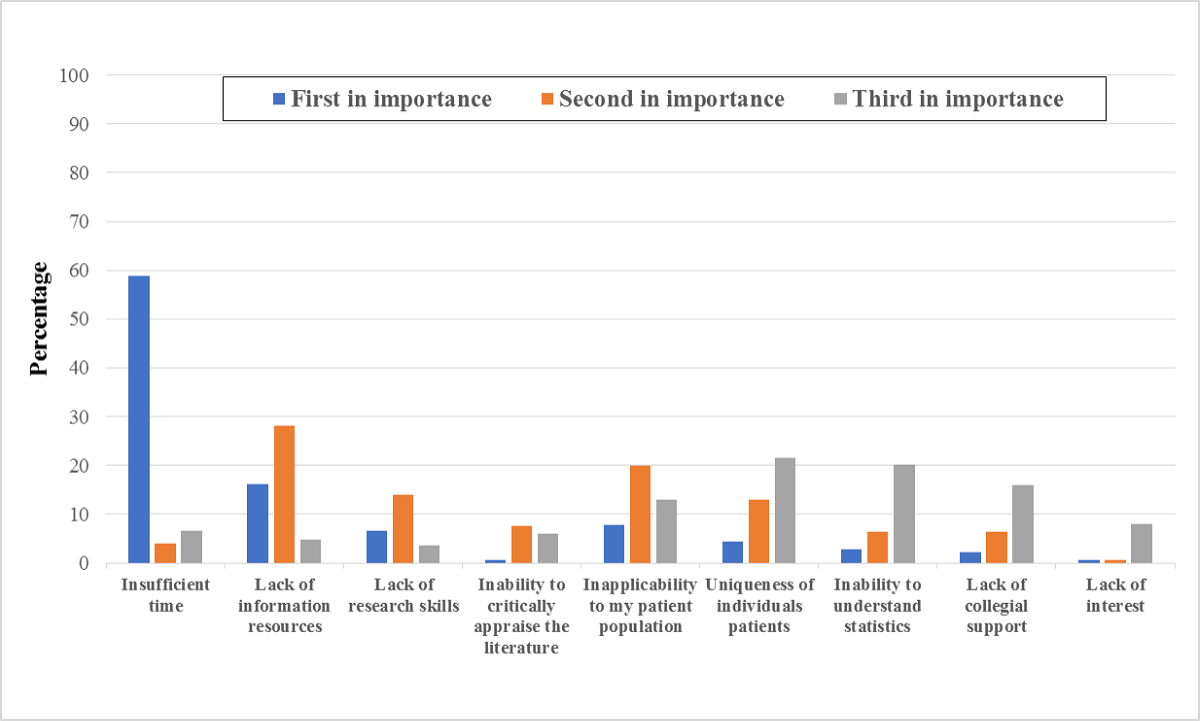
Self-reported ranking of barriers to evidence-based practice.

Almost 59% of the PTs ranked lack of time as the greatest barrier to the use of EBP in their practice, with approximately 70% indicating that this factor was one of the top 3 barriers. Lack of information resources (49%) and inapplicability of research findings to their patient population (40.6%) were the second and third greatest barriers, respectively (Figure 6).

### Results From the Semistructured Interview

#### Participant Description

All but 2 of the interviewees were female. The participants held BSc degrees in PT, except the head director, who held an MSc degree in PT. The age of the interviewees ranged from 40 to 50 years.

#### Interview Themes

##### Background, Knowledge, Attitudes, and Skills for Evidence-Based Practice

The interview results revealed that 80% of the PT directors had heard about EBP and 60% attended EBP workshops. All of the directors showed willingness to adopt EBP into their PT practice and emphasized the importance of offering continuous training courses or workshops on this topic. These directors believed that PT is primarily evidence based, and 1 director stated the following: “All of the physical therapy techniques that are used for patients are evidence based.”

With regard to the knowledge and skills required to practice PT based on evidence, 1 director indicated that enthusiastic PTs should have the knowledge and skills needed to search, find, and apply the best and most up-to-date treatment techniques and stated the following: “We have some specialists who are truly active in their practice and are ready to do the best job for the better care of patients—most are new graduates from BSc programs or hold MSc degrees or PhDs.”

**Table 5:** Factors (demographics) associated with access to and availability of the literature. The number of respondents varies because of missing data.

Terms and factor			Odds ratio (95% CI)	Model *P*^a^ value	Model *R*^2^^b^
**Access to paper journals**					
	**Work settings (n=** **181)**			<.001	.188
		General hospital	1.154 (0.508-2.622)		
		Rehabilitation hospital	0.105 (0.028-0.396)		
		Specialized hospital	Reference		
**Relevant practice guidelines available (n=170)**					
	**Working hours/week**			.014	.121
		<10	0.330 (0.095-1.142)		
		10-19	0.231 (0.032-1.680)		
		20-29	5.538 (0.636-48.262)		
		30-39	0.699 (0.238-2.058)		
		40+	Reference		
**Access to Web-based practice guidelines**					
	**Gender** **(n=176)**			.02	.044
		Male	2.288 (1.127-4.646)		
		Female	Reference		
**Access to Web-based databases at work**					
	**Work settings (n=181)**			.001	.099
		General hospital	2.242 (1.003-5.009)		
		Rehabilitation hospital	0.587 (0.245-1.411)		
		Specialized hospital	Reference		
**Access to Web-based databases away from work**					
	**Age (years; n=182)**			.02	.082
		20-30	2.231 (0.604-8.243)		
		31-40	6.091 (1.662-22.324)		
		41-50	4.556 (1.190-17.433)		
		51+	Reference		
	**Area of specialty (n=177)**			.004	.109
		Neurology	0.165 (0.042-0.653)		
		Cardiopulmonary	0.244 (0.058-1.022)		
		Orthopedics	0.648 (0.171-2.457)		
		Pediatrics	Reference		

^a^In logistic regression, one level of the independent variable serves as a reference against which the odds of the other levels occurring are determined.

^b^Nagelkerke *R*^2^.

##### Opinions on Implementing Evidence-Based Practice

All of the interviewees agreed on the importance of EBP in PT, as applying the latest and best therapy for patients is the main aim in health care. However, the interviewees believed that EBP in PT relies on staff readiness. Of them, 1 of the interviewees discussed the importance of EBP with her staff and received this response from a staff member: “It is an unimportant addition to our practice, and we do not need to go for it”.

All of the directors underlined the need for EBP in their practices and to look for the best way to incorporate EBP into routine work. Of them, 1 director stated the following: “PT is not a stressful profession, and we are not working under pressure, so it’s feasible to practice EBP to provide better care.”

##### Barriers and Facilitators to Adopting Evidence-Based Practice in the Physical Therapy Field

The interview results show that there were 2 main barriers to EBP in the PT departments: (1) staff resistance because of a lack of interest or unwillingness to do extra work and (2) busy staff schedules.

Most of the interviewees (80%) mentioned that some staff made individual efforts to conduct Web-based searches on the literature reviews using their personal laptops and Wi-Fi connections. Furthermore, all of the interviewees mentioned the need for workshops to provide staff with the knowledge and skills needed for EBP in PT. Of them, 1 director stated the following: “a lack of knowledge and skills is considered a barrier.”

The interviewees pointed out that the atmosphere of the work environment has a positive influence. However, they stated that the culture of the organization and attitudes of the patients do not influence adopting a new technique of therapy. Most of the interviewees (90%) confirmed that the key factor when selecting and applying the best therapy is the capability of the practitioner to communicate with the patient effectively. The importance of communication skills for PTs was stressed by most of the interviewees. Of them, 1 individual said: “We always rely on our communication skills to convince patients to take the recommended PT, and these skills are required for applying new techniques, as in the case of EBP.”

All of the interviewees agreed on the importance of the support from upper management and stakeholders to implement EBP, such as the financial support needed for providing computer labs and access fees required for medical databases. Of them, 1 of the interviewees commented: “I think we need a formal decision from the upper management to go for EBP so that all facilities required would be offered accordingly.”

## Discussion

### Overview

In general, the PTs in this study had positive attitudes and showed interest in learning or improving the skills necessary to incorporate EBP into their practice. Their educational background, knowledge, and skills related to accessing the literature, interpreting the research evidence, and applying this information to therapeutic intervention practices were well-founded during their undergraduate education. However, their access to and the availability of electronic resources to implement EBP, together with the support to use such resources at work, were lacking.

### Positive Attitude, but Not the Beliefs

Despite the overall positive attitude of PTs and their interest in EBP, which was in agreement with several previous studies [6-13], some PTs in this study were skeptical in some of their beliefs. They believed that EBP might create unreasonable demands on them, particularly the PTs who were working more hours per week. The PTs also believed that strong evidence was not available to support most of the interventions they used with their patients. This belief was more persistent among PTs working in the general hospital than the belief in those working in the rehabilitation or specialized hospitals. A possible explanation for this difference might be the variety of cases seen in the general hospital. Another possible explanation might be the lack of resources, a reported barrier toward adopting EBP in this study, which in turn might limit the access of PTs to electronic databases that would offer necessary research evidence. These reported uncertainties were consistent with previous studies reporting that the unavailability of evidence [10,11,14], busy schedule, and lack of time inhibited EBP [6-8,10,11,15,16]. Finally, the PTs in this study believed that EBP does not consider patient preferences. This belief is probably because of their lack of understanding of the 3 principle elements of EBP: the best available and most up-to-date research evidence, the clinical experience of the health care providers, and the values and preferences of the patient at any stage of the medical care process [22]. Thus, it appears that the PTs in this study thought that EBP is concerned only with the use of the best available and most up-to-date research evidence and ignores the preferences of patients. This feeling was evident during the interviews with the directors, as 1 director criticized the demand for EBP and claimed that their daily practice was already based on evidence. This belief also points to the lack of communication with the patients. Hence, PTs should communicate effectively with and address the preferences of patients to incorporate new evidence regarding therapies [23]. The results of the interviews reinforce this notion and support the importance of communication skills in EBP during PT treatment.

### Educational Background, Knowledge, and Skills

In this study, the PTs had learned the basics of EBP during their undergraduate education, including the knowledge of the statistical terms and the skills necessary to access and interpret information from the literature. This finding agrees with the findings of a previous international study [12]. In addition, findings from the interviews show that newly graduated PTs or PTs with postgraduate degrees were active and could contribute positively to EBP to provide the best PT care. Thus, it is reasonable to infer that the majority of the PTs in this study had the competency to adopt EBP. However, knowledge of some advanced statistical terms, such as *odds ratio*, *meta-analysis*, *confidence interval,* and *heterogeneity*, was deficient among the PTs. These findings reveal the need for the continuous participation of PTs in training courses or workshops to expand their knowledge. The qualitative results support this finding, wherein the PT directors asked for workshops, local or abroad, for preparing qualified evidence-based practitioners.

### Administrative and Technical Support at Work

At work, the PTs in this study had insufficient administrative support to use EBP and limited access to information resources to promote EBP. In addition, lack of time, lack of information resources, and inapplicability of research findings to specific patient populations were reported. These findings were consistent with several previous findings [6-8,10,11,14-17]. Similarly, the interview findings in this study showed similar barriers against implementing EBP. Furthermore, cultural factors had no influence on adopting EBP in PT, as the health care system in the State of Kuwait focuses on patient care, and this finding was confirmed in a previous study [5]. However, because of a lack of higher management support in terms of providing the facilities required (eg, electronic libraries) and encouragement for EBP or the lack of interest or unwillingness of some PTs to adopt new tasks in their busy schedules, staff resistance might arise.

### Implications of Findings

On the basis of the study findings and interviews, several solutions emerged. Multimedia Appendix 2 lists the possible barriers and solutions. The solutions are relevant to the directors, staffs, and patients, locally and to countries with a similar work culture.

### Strength and Limitations

This study was the first to be conducted among PTs from all specialties in government hospitals in Kuwait. Taking the high response rate into account (92%), the results can be considered to represent the target population of PTs across government hospitals. However, this study did not include PTs working in private hospitals or clinics. Thus, the findings cannot be generalized beyond the study sample. Although the questions used in the study questionnaire were derived from previously validated and reliably tested tools, the validity and the reliability of this questionnaire were not tested in this specific population. Furthermore, this study relied on self-reported data; thus, the PTs might have over reported some information. However, the qualitative results support the overall findings in several ways.

### Conclusions

The PTs in this study lacked support at work and were in need of a continuous education program to expand their knowledge after graduation. Thus, identifying methods and strategies to support PTs in adopting EBP in the State of Kuwait is necessary.

## Appendix

Multimedia Appendix 1 Interview questions.

Multimedia Appendix 2 Barriers and solutions.

## Abbreviations

BSc: Bachelor of Science
EBP: evidence-based practice
KMLA: Kuwait Medical Licensing Authority
MoH: Ministry of Health
MSc: Master of Science
PTs: physical therapists
PT: physical therapy
