# Supplementary material for: Evidence-Based Physical Therapy Practice in the State of Kuwait: A Survey of Attitudes, Beliefs, Knowledge, Skills, and Barriers
Source: JMIR Med Educ. 2019 Jun 7;5(1):e12795. doi: 10.2196/12795 (PMC6682286; doi:10.2196/12795)
Supplement: Multimedia Appendix 1 [file mededu_v5i1e12795_app1.pdf]

Appendix 1: Interview questions

**1.** Demographic information
• Including participant’s age, gender, education, and years of experience
**2.** Background, knowledge, attitudes, and skills for EBP
• Have you heard about EBP? Attended any workshop?, seminar?, etc.<br>• Are you willing to adopt EBP into PT?<br>• What are the staff abilities in order to practice this approach?<br>• Knowledge and skills required for EBP
**3.** Opinions on implementing EBP
• What do you think about the importance of the need for evidence-based PT?<br>• Is PT staff ready for this evidence-based practice?
**4.** Barriers and facilitators to adopting EBP in the physical therapy field
• What are the factors that obstacle the adoption of EBP in the PT field?<br>• Staff resistance, such as lack of interest, or lack of knowledge and skills for EBP. Moreover, the influence of the culture of the organization on adopting new technique for PT<br>• What are the factors that facilitate the adoption of EBP in the PT field?<br>• Upper management support, physical requirement, such as computer station, internet connection, and free access to medical databases
