# Supplementary material for: Evidence-Based Physical Therapy Practice in the State of Kuwait: A Survey of Attitudes, Beliefs, Knowledge, Skills, and Barriers
Source: JMIR Med Educ. 2019 Jun 7;5(1):e12795. doi: 10.2196/12795 (PMC6682286; doi:10.2196/12795)
Supplement: Multimedia Appendix 2 [file mededu_v5i1e12795_app2.pdf]

Appendix 2. Barriers and solutions

Barrier	Solutions
Busy staff schedule	• Time management (e.g., assigning less work to two PTs so that they can perform EBP tasks)<br>• A formal decision from the upper management to authorize and include EBP in PT
Lack of communication skills of some PT specialists	• Improve communication skills with medical staffs, patients, and academic PTs<br>• Run awareness campaigns to improve collaboration among medical staff and with patients to increase patient confidence
Lack of motivation	• Offer incentives at early stages<br>• Hold awareness sessions to motivate staff and increase their willingness to provide the best care to patients
Need for proper management	• Offer continuous support<br>• Gradually apply new practices in PT to ensure accuracy, completeness, and continuance
Patient attitudes	• Develop awareness guidelines about EBP for patients and disseminate them through bulletin boards, brochures, and roll-up banners.<br>• Involve patients in the treatment process.
Lack of interest in adding new practices	• Organize awareness sessions for staff to highlight the positive outcomes of EBP<br>• Arrange workshops, local or abroad, for those staff who are willing to gain EBP knowledge and skills
